# Yellow Fever Plague

**Published:** 1878-09

**Authors:** 


### The Yellow Fever Plague.

The appearance of yellow fever in a malignant form at New Orleans and other points in the Gulf states, has naturally caused much anxiety on the part of the sanitary and health officers of other localities for protection from it. As this is a portable disease, not only sea-board but also interior cities having water and railroad communications with that section are vitally interested.

The history of the origin of this outbreak whether from recent importation or developed from an old fomites hidden away, which had preserved its vitality in some obscure place where the disease had once prevailed, and again revived its latent poison under favorable condition, or originating *de novo*, we shall not discuss. We will here quote a few sentences relative to the natural home of yellow fever, from a paper by Dr. J. M. Toner, entitled "The Distribution and Natural History of Yellow Fever, as It Has Appeared at Different Times in the United States," read before the American Public Health Association in the city of New York in the fall of 1874, and published in the report of the Surgeon General of the marine hospital service of the United States for that year, with a map showing the localities in the United States where the disease has appeared from 1668-1874: "The conceded home of yellow fever is in the West Indies and the Bahamas, with a portion of the adjacent continents of North and South America. A square formed by the 45th and 100th degrees of longitude, and the 35th north and the 5th south latitude, will include the favorite region of this disease. Although originating within the square named, history shows that it may prevail on the sea-coast in any locality within the tropics, north and south of the equator, where malarial fevers prevail, and the daily average of the thermometer is over 75 or 80 degrees, with a high dew-point for weeks or months together." There are, no doubt, other conditions than those of continued high temperature with moisture of a purely sanitary and preventative character, which favor the existence and propagation of this great scourge of the tropics. It has been observed that once the disease becomes epidemic it can exist and preserve its grave form at a much lower temperature than seems to be required for its origin. Yellow fever of a malignant type has appeared very early this season in the southern part of the United States, and is spreading with unexampled rapidity and mortality to the cities in the gulf states. It is also advancing steadily up the Mississippi valley into the heart of the country. In the past it has appeared in most of the Atlantic maritime and coast cities as far north as Boston, and indeed has been chronicled at Quebec and Halifax. The history of the geographical limit of the disease in an epidemic form shows its northern boundary to be in the region of Norfolk, Va. This disease prevails in warm, and in cities rather than in rural places. It is sometimes arrested in its course or modified in its severity by storms, heavy and continued rains. The polar waves or "northers" that occasionally blow from the Arctic region down across Texas have been known to almost completely arrest its severity and spread. We cannot now reasonably expect the complete arrest of this epidemic until the advent of frost, which always effectually puts an end to it for the season.

While it is true that yellow fever has visited many of the seaport cities, it has, fortunately, never extended to the interior of our country, away from the towns and cities located along the great water courses to which it was carried. Dr. Drake many years ago observed that while the disease had appeared at almost every town on the Mississippi, as far up as Vicksburg, that Woodville, twelve miles from the river, was the most remote inland point it had reached. The changed condition of our country, in its development of railroads, with the growth of villages into towns, and towns into cities, have rendered it possible for this disease to be carried within a few hours or a day or two at most to any of our interior cities, and where it is probable such unsanitary conditions exists that it could propagate itself during the heated term. Once the disease has become epidemic in a city the unacclimated have no security except by flight to healthy localities.

There are, perhaps no cities out of the Gulf states where the citizens could be said, in any sense, to be acclimated to yellow fever.

